# Nano-Synthetic Devices in Leishmaniasis: A Bioinformatics Approach

**DOI:** 10.3389/fimmu.2015.00323

**Published:** 2015-06-19

**Authors:** Milsee Mol, Dipali Kosey, Shailza Singh

**Affiliations:** ^1^National Centre for Cell Science, Savitribai Phule Pune University Campus, Pune, India

**Keywords:** *Leishmania*, synthetic biology, nanotechnology, bioinformatics, nanodevices

## Abstract

Synthetic biology is an investigative and constructive means of understanding the complexities of biology. Substantial progress in the fields has resulted in the creation of synthetic gene circuits, which when uploaded into the appropriate nanoliposomal vehicle, can be used for a tunable response in a cell. These tunable elements can be applied to treat diseased condition for a transition to a healthy state. Though in its nascent stage of development synthetic biology is beginning to use its constructs to bring engineering approaches into biomedicine for treatment of infectious disease leishmaniasis.

In the twentieth century, Feynman’s “There is plenty of room at the bottom” lecture for the meeting of American Physical Society at Caltech (1959) opened a firework of intellectual concepts to a wide range of themes within physics and wherever physics was involved. This lecture laid the foundation of creating nano sized entities that could be useful for human welfare. Alongside, people in the creative industry were coming up with crazy stories that brought up the Hollywood science fiction movie *Fantastic Voyage* in 1966 with illustrations of nanorobots with medical abilities. These could excavate plaque in clogged blood vessels, locate pathogens, and inject materials into the red blood cells. These conveyed that nanomedicine could be the “next” big step to cure diseases with precision (1). But it was only with the invention of scanning tunneling microscope in 1981; almost 15 years of Feynman’s Caltech lecture that nanotechnology emerged as a major research discipline. Since then nano sized materials have made their impact in almost all facets of human life; of particular mention is its contribution to medicine. Man-made nanoscale systems match the scale of the physiological working levels in biology and therefore have great relevance to its application in the field. A variety of nanoscale materials are being used in medical and clinical research as detectors, targeted drug delivery vehicles, in drug screening, imaging, and diagnosis (2). With advances in nanotechnology, today’s nanomedicine come packed in surface modified nanocarriers having immense therapeutic potential due to their targeted action and biocompatibility; and are available in the market (3) (Table 1A). Also previously inaccessible areas of the body can be made accessible by propelling the nanodevices by a variety of fuel and fuel free driven technologies to deliver therapeutic and diagnostic agents (4) (Table 1B).

**Table 1:** **(A) Nanomedicines; (B) Propelling systems applied to nanodevices; (C) Biomolecules as nanostructures in therapeutics**.

**(A) NANOMEDICINES AVAILABLE IN THE MARKET AND THEIR INDICATIONS**			

**Drug**	**Nano carrier**	**Application**	**Brand name**

Paclitaxel	Albumin-bound nanoparticles	Metastatic breast cancer	Abraxane^®^
Doxorubicin	Liposomes	Metastatic breast cancer	Myocet^®^
Doxorubicin	PEGylated liposomes	Metastatic breast and ovarian cancer; Kaposi sarcoma	Caelyx^®^
Daunorubicin	Liposome	Kaposi sarcoma	DaunoXome^®^
Amphotericin B	Liposome	Fungal infections and cutaneous leishmaniasis	AmBisome^®^
Monoclonal sheep antibody	PAMAM dendrimers	Cardiac marker diagnostic	Stratus^®^

**(B) PROPELLING SYSTEMS**			

**Propulsion mode**	**Nano carrier**	**Application**	

Catalytic	Multilayer microtubes (5)	Immuno-micromachine-based approach for *in vitro* detection circulating tumor cells without sample preprocessing (6)	
Magnetic	Tumbling nanowires (7)	Magnetic resonance guided microcarrier for liver chemoembilization (8)	
Ultrasound	Mutilayer microtubes (9)	Acoustically active microbubbles for effective therapies in primary central nervous system lymphoma (10)	

**(C) BIOMOLECULES AS NANOSTRUCTURES IN THERAPEUTICS**			

**Biomolecule**	**Application**		

Proteins	Self assembly nanopeptide vaccines to display B-cell epitope from the malaria parasite *Plasmodium berghei* circumsporozoite protein (20)		
RNA	Transferrin-tagged, cyclodextrin-based polymeric nanoparticles-CALAA-01, contains siRNA that targets the M2 subunit of ribonucleotide reductase for cancer treatment (21)		
	HIVgp120: tat-rev siRNA chimera, a 16-nt dsRNA used as the scaffold to link the gp120 aptamer; decreases infection by blocking gp120 and prevents replication by silencing tat-rev (22)
DNA	Cancer-fighting DNA nanorobots that target specific cells for repair (23)		

Nanotechnology to medicine is becoming more promising as biocompatible materials like proteins, RNA, and DNA (Table 1C) are being molded into nano cargoes with targeting scaffolds for targeted delivery systems (11–18). These natural polymers have the tendency to self-assemble into versatile nanostructures using the information encoded within their defined sequence. Of all these DNA seems to be superior as it has the structural and functional information encoded within it and a variety of DNA manipulating enzymes provide the much needed tool box required to design a myriad of DNA nanostructures (16). DNA via controlled assembly can be nano-engineered to one-dimensional (1D), two-dimensional, and 3D DNA nanostructures. DNA hybridization to short “stapler” DNA is a versatile method to construct DNA origami nanostructures (18). Proteins and RNA are genetically encoded into DNA sequence and therefore DNA sequences encoded with desired nano functionality can be regarded the “one in all” biocompatible material for expressing nanostructure within a cellular chassis. It can be regarded as an aspect of synthetic biology, as using defined DNA sequences, hybrid (protein–RNA–DNA) nanostructures can be designed and fabricated that do not exist in nature; idealizing with the mainframe concept of synthetic biology. This will not only add to the wide range of functionalizing the nanostructure but also will aid in the multistage nanoassembly systems (19). Thus, we can say that DNA has come a long way from just being an entity to be studied in genetics to a multimodal nanomachine with wide applications in bionanomedicine.

## Bioinformatics Approaches Applied to BioNanotechnology

Post Genomics Era, Bioinformatics has taken the center stage in storing, managing, visualizing, and retrieval of loads of data that can be systematically dealt for understanding the complexities in biology. Similarly, the information generated in nanotechnology should be catered to by developing new bioinformatics and computational tools that may be critical in designing, modeling, simulation, and visualization of bionanosystems that may arise with amalgamation of synthetic biology and nanotechnology. As of now, nanotechnology is relatively new in biology and the data that comes from experiments and modeling studies is scattered. It is only recently that efforts like the Investigation Study Assay (ISA) tab-delimited (TAB) format (ISA-TAB-Nano) (24), Cancer Nanotechnology Laboratory (caNanoLab) (25) are being directed to build databases for nanotechnology derived data. Curation of the data in nanotechnology databases is important as experimental conditions to obtain the nanomaterial and its application can affect the actual data measurement. Nanomaterial registry (26) has developed a method for combinatorial analysis and curation of these datasets for characterized nanomaterials. Similarly, tools for visualization, molecular modeling, docking, QSAR, and Designing Biomolecular nanostructures are available (27).

For synthetic biology assisted nanotechnology, the major advantage of an advanced computational tool will be aiding and conceptualizing novel nanodesign with the option of exploring many structural options and their stability statistical analysis (28, 29). The raw sequences required for nanostructure design can be culled from the many databases available in the public domain like the GenBank, EMBL, UniProt, RNACentral, etc. These raw sequences can be further modified as per the demands of the structural determinants of the conceptualized bionanostructure. Further statistical analysis of these predicted structure helps in validating their stability, robustness, and evolvability. Many state of the art mathematical packages are available like MATLAB^®^ and Bioconductor that can perform various statistical analyses on the given dataset.

Synthetic-DNA nanotechnology must be applied to infectious disease like leishmaniasis. Leishmaniasis is one of the most neglected tropical diseases of the world, killing almost 20,000–300,000 people in the developing countries. Many drugs have been made and are still being discovered, but the protozoan re-emerges showing drug resistance to the effective medications. Nanotechnology has just managed to show its promise in developing a liposomal formulation called amphotericin B for leishmaniasis, which is available in the market, but has serious side effects and is not cost effective in developing countries (30). Initial, nanodelivery systems for delivering chemotherapeutics were nanodisks impregnanted with amphotericin B, polymeric-nanoparticle loaded with pentamidine, primaquine, and niosomes (31), which still need validation at the clinical level. There is an urgent need to take up assignments to use effective nanotechnology devices in combating this infectious disease. DNA nanostructures have shown promise in treating cancer and many of them very well could come into clinical trials in the near future. But a lot needs to be done as far as infectious diseases are concerned. DNA nanotechnology and its potentials can be tapped in infectious disease for immunostimulation and vaccine preparation.

## Nanotechnology for Leishmaniasis

*Leishmania* are susceptible to complement-mediated lysis during the promastigote stage of their life cycle. This form of the parasite is found immediately after injection into the host’s blood stream by a sandfly vector. These parasites can coat themselves with C3b complement that act as opsonins to gain entry into macrophages where they can survive intracellular killing. These brief window periods of complement susceptibility could be exploited to kill parasites with aptamers by coupling to complement-mediated lysis (32). Aptamers can be linked to DNA or proteins that confer polyvalent functionalities, one being cell internalizing capability that only targets a particular cell population and deliver their therapeutic cargos specifically into the cell, in leishmaniasis the promastigotes, this may enhanced therapeutic efficacy and reduce cellular toxicity. Also, DNA nanoscaffolding of aptamers may make them resistant to serum nucleases and impart lower immunogenicity.

Macrophage M1 to M2 differentiation is the bottleneck for a proinflammatory response in many of the infectious disease. In leishmaniasis too, since the protozoan resides within the macrophages, it blocks its transformation to M2 type and silences the immune alarming system for an inflammatory response deciding the pathogenesis in disease outcome. Self-assembled nanostructures with that extracellular matrix (ECM) mimicking capability may also display biological signals together, which can help modify and instruct cell behavior. Structurally, programmable nanostructures can also modify signaling components and DNA nanostructures as artificial scaffolds can be used to control cell behavior. If these DNA nanostructures can be coassembled with peptides having biological epitopes for cell receptors that can act as signal for cell differentiation (33). Such co-assemblies investigation formed could be capable of guiding the differentiation of M1 to M2 macrophages.

The leishmanial parasite PAMPS mainly the LPGs are recognized by TLR2 and TLR4, which initiates an initial but a very short time spanning TLR-mediated inflammatory response. But using the many survival strategies the parasite affects the TLR signaling and subverts the inflammatory response. The endosomal TLRs can come to the rescue, if they are activated with appropriate signal for disease resolving response. CpG nanostructures directed to the endosomes can be utilized for the activation of innate immune response. The endosomal TLR3, TLR7/8, or TLR9 recognizes exogenous or endogenous RNA/DNA in endosomal vesicles. Bacterial and viral DNAs harbor CpG motifs with much higher frequency compared with mammalian DNA and have the capability to activate the endosomal TLR signaling (34). The activated TLR9 in macrophages can then secrete proinflammatory cytokines and type I. Soluble DNA nanostructures can be used for the targeted delivery of CpG DNA to TLR9-positive cells targeted to the macrophages harboring the leishmanial parasites.

*Leishmania* also has the capability to inhibit apoptosis of the resident macrophages. Apoptosis can be induced in the macrophages by using the biomimetic material platform composed of self-assembling hybrid nanoconjugates as a therapeutic system. Apoptosis is induced by clustering of CD20 within lipid rafts. If these CD20-bound antibodies are hyper-cross-linked by Fc receptor (FcR) on macrophages they can be made to undergo apoptosis. These are termed as “drug-free macromolecular therapeutics” due to the absence of low-molecular-weight drugs that often show side effects (35). In this way, apoptosis induction could be made direct and specific toward the innate immune response for intracellular killing of the leishmanial parasite.

The leishmanial parasite is known for its ability to modulate the innate immune signaling for its safe intracellular survival. DNA–protein nanoscaffolds can be used for modulators of immune signaling with in the macrophages. Earlier work have used RNA as scaffolding device within bacterial cells with capabilities for increased H2 production by varying the relative geometries of the enzymes with in the cell (36). Through rational design, both DNA and RNA can be used to create 1D, 2D, and 3D structures, allowing stable and rigid arrangements of enzymes for favorable substrate channeling (37). Similarly, in leishmaniasis, the innate immune signaling can be channelized for a proinflammatory response by orienting the signaling molecules using DNA–RNA scaffolding machinery.

Vaccine development in leishmaniasis is another major area where DNA nanotechnology may have an important contribution. Till date, many efforts have been undertaken to develop a successful vaccine, but realizing them into the clinics has been a bottle neck. Whole cell killed, attenuated, subunit vaccines have been developed in the past. Liposomal soluble *Leishmania* antigen incorporated CpG has also been tested in mouse model of cutaneous leishmaniasis, and generated significant levels of protection. Recombinant proteins using virus as a delivery vehicles have also been tested as vaccines in preclinical studies (38). Recent efforts are being made to assemble multiple adjuvant elements on a DNA nanostructure, increasing their immunostimulation capacity, highlighting the potential of DNA nanostructures to serve as new platforms for vaccine construction. Tetrahedral DNA nanostructure has been used as scaffold to assemble a model antigen, streptavidin (STV), and a representative adjuvant, CpG oligo-deoxynucleotides (ODN), into a synthetic vaccine complex, which elicits antibody response against the model antigen, STV. Programmable DNA nanostructures provide an excellent platform for construction of vaccines with multivalency and three-dimensional configuration. The three-dimensional arrangement of each of the immunogenic components can be readily controlled through the rational design of the scaffold sequences (39). Strong antigenic proteins from leishmanial parasite can be picked up for conjugation with DNA nanostructures for vaccine development (40).

**Figure 1:**
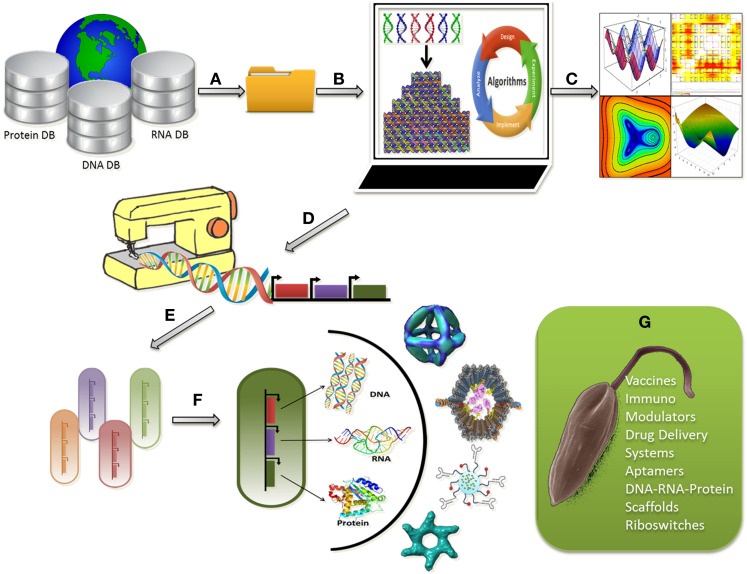
**Programmable synthetic constructs can be used to assemble nanostructures within living cells in a controlled fashion for leishmanicidal effects;** **(A)** Raw sequence data of proteins, DNA, and RNA can be retrieved from databases; **(B)** Raw sequence data analysis for selection of a good sequence for nanostructure design followed by designing, modeling, simulation, and visualization of bionanosystems; **(C)** Computational analysis of the nanostructures for stability, robustness, and evolvability; **(D)** Generating sequence for tailor made nano-synthetic constructs; **(E)** Selection of a suitable chassis microorganism for uploading the synthetic construct; **(F)** Optimum chassis uploaded with the construct for the synthesis of combinatorial nanostructures; **(G)** Application of the nanostructures in the form of vaccines, aptamers, immune modulators to leishmanial parasite for a leishmanicidal effect.

Taken together, understanding the parasite–host interaction at the systems level and picking up the nuances in the interaction network may aid synthetic device construction. A combinatorial treatment strategy, with available drugs and novel nanostructures, may be designed rationally for the treatment of leishmaniasis. Such strategies may help overcome drug resistance and act as supportive mechanism to block the functional redundancies serving as an escape route for parasite survival (Figure 1).

## Challenges: Computation in Nano-Synthetic Biology for Leishmaniasis

Since the first genome of *Leishmania* species was released in 2005, four more species have been sequenced (39) and databases like GeneDB and LeishCyc are available, which store data pertaining to the genome and metabolic pathways in *Leishmania*. Nanotechnology being applied to treat leishmaniasis is a relatively new area of research and a lot still needs to be done to set up merge the available datasets with new data that will arise when nanotechnology will be applied to various aspects of the leishmanial disease. Due to lack of information, the major research challenges are

- Finding new reference nanomaterials using proteins, RNA, and DNA against the leishmanial parasite
- Improved characterization of these nanomaterial
- Standardizing the experimental setup with reproducible sensitivity and accuracy
- Quantify and negate any error in protocols used to produce the data.

Nanotechnology when applied to leishmaniasis may raise numerous challenges due to the complexity associated with the life cycle of the parasite, its interaction with the host, its genome annotation are still not delineated. Moreover, significant investment in informatics is required to accelerate current research in understanding such a complex protozoan disease. Though *Leishmania* post-genomic research projects, have transformed the way the parasite is treated, for nanotechnological application, computational models, and algorithm for simulations of predicted annotated genome of *Leishmania*, needs a new approach.

As we move toward understanding the complexities associated in a diseased condition, better are the chances of discovering novel concepts and targets for drug delivery and treatment. We envisage that nano-synthetic biology might revolutionize the current biomedical science and the treatment technology in future making the illustrations of the *Fantastic Voyage* a reality.

## Conflict of Interest Statement

The authors declare that the research was conducted in the absence of any commercial or financial relationships that could be construed as a potential conflict of interest.

## Abbreviations

caNanoLab, cancer nanotechnology laboratory; ECM, extra cellular matrix; ISA-TAB-Nano, investigation study assay tab-delimited format; ODN, oligo-deoxynucleotides; PAMAM, poly (amidoamine); PAMP, pathogen associated molecular patterns; PEG, polyethylene glycol; QSAR, quantitative structure–activity relationship; SNAPL, stable nucleic acid lipid particle; STV, streptavidin; TLR, toll like receptors.
